# Practical guidance on the clinical management of ocular adverse events associated with belantamab mafodotin in patients with relapsed/refractory multiple myeloma: Recommendations from a Japanese expert panel

**DOI:** 10.1093/jjco/hyaf148

**Published:** 2025-10-06

**Authors:** Kazutaka Sunami, Tomoaki Fujisaki, Toshinari Funaki, Michiko Ichii, Shigeki Ito, Morio Matsumoto, Koh-ichi Oshima, Kazuhito Suzuki, Teruhito Takakuwa

**Affiliations:** Department of Hematology, NHO Okayama Medical Center, 1711-1 Tamasu, Kitaku, Okayama 701-1192, Japan; Department of Internal Medicine, Matsuyama Red-Cross Hospital, 1 Bunkyocho, Matsuyama, Ehime 790-8524, Japan; Department of Ophthalmology, Japanese Red Cross Medical Center, 4 Chome-1-22 Hiroo, Shibuya, Tokyo 150-8935, Japan; Department of Hematology and Oncology, Osaka University Graduate School of Medicine, 2 Chome-2 Yamadaoka, Suita, Osaka 565-0871, Japan; Hematology and Oncology, Department of Internal Medicine, Iwate Medical University School of Medicine, 1 Chrome-1 Idaidori, Yahaba, Shiwa, Iwate 028-3694, Japan; Department of Hematology, NHO Shibukawa Medical Centre, 383 Shiroi, Shibukawa, Gunma 377-0204, Japan; Department of Ophthalmology, NHO Okayama Medical Center, 1711-1 Tamasuku, Kita, Okayama 701-1192, Japan; Division of Clinical Oncology/Hematology, Department of Internal Medicine, The Jikei University School of Medicine, 3 Chome-25-8 Nishishinbashi, Minato, Tokyo 105-8461, Japan; Department of Hematology, Wakakusa Daiichi Hospital, 1-6 Wakakusacho, Higashiosaka, Osaka 579-8056, Japan

**Keywords:** multiple myeloma, cornea/drug effects, Japan, drug-related side effects and adverse reactions

## Abstract

**Background:**

Multiple myeloma is a significant cause of mortality, and treatments for patients with relapsed/refractory multiple myeloma have limited efficacy. Treatment regimens with belantamab mafodotin have demonstrated significant improvement in progression-free survival compared with current standard-of-care regimens but have been associated with an increased risk of ocular adverse events (OAEs). These practice guidelines aim to provide recommendations to support Japanese clinicians in managing OAEs and facilitate confidence in the use of belantamab mafodotin.

**Methods:**

An expert panel of Japanese haematologists/oncologists and ophthalmologists convened to discuss and agree on the recommendations for managing OAEs in patients treated with belantamab mafodotin.

**Results:**

The expert panel identified four key themes and 13 clinical questions to guide the recommendations for managing belantamab mafodotin–related OAEs in the real-world setting in Japan. The four key themes were: (i) identification of OAEs associated with belantamab mafodotin treatment; (ii) management and dose modification of belantamab mafodotin treatment for OAEs; (iii) multidisciplinary collaboration for effective management of ocular events; and (iv) a patient-centred approach to the management of OAEs.

**Conclusions:**

The recommendations in these practice guidelines build on published clinical trial evidence and the practical experience of the expert panel to help Japanese clinicians make informed treatment decisions in the management of multiple myeloma in the real-world setting.

## Teaser

Belantamab mafodotin–based regimens demonstrated efficacy in patients with relapsed/refractory multiple myeloma, but with increased risk of ocular adverse events. These guidelines will help patients and clinicians in shared decision-making.

## Introduction

Over 187 000 new cases of multiple myeloma (MM) were reported worldwide in 2022, with over 121 000 deaths due to MM also reported [1]. In Japan, there were an estimated 8000 new cases of MM and 4500 MM-related deaths in 2023 [2]. Patients with newly diagnosed MM (NDMM) are typically treated with triplet or quadruplet combination regimens, including an immunomodulatory agent, a proteasome inhibitor, and/or an anti-CD38 monoclonal antibody [3]. In Japan, patients with NDMM are increasingly treated with daratumumab + lenalidomide + dexamethasone, which may lead to an increase in the number of patients refractory to these treatments at second line and beyond [4]. Because the likelihood of response and duration of response decreases with each subsequent line of therapy [5], treatments with novel mechanisms of action are required for patients with relapsed/refractory MM (RRMM).

Belantamab mafodotin is an antibody–drug conjugate (ADC) comprising a humanized anti-B-cell maturation antigen (BCMA) monoclonal antibody conjugated to the microtubule inhibitor, monomethyl auristatin F [6]. Recently, two phase 3 clinical trials have demonstrated a significant and clinically meaningful benefit of belantamab mafodotin combinations: in DREAMM-7, belantamab mafodotin, bortezomib, and dexamethasone resulted in significant improvement in median progression-free survival (PFS) compared with daratumumab, bortezomib, and dexamethasone (36.6 months vs 13.4 months, respectively) [7]; in DREAMM-8, belantamab mafodotin, pomalidomide, and dexamethasone significantly improved PFS compared with pomalidomide, bortezomib, and dexamethasone (median PFS not reached vs 12.7 months, respectively) [8].

Ocular adverse events (OAEs) were reported in 79% and 89% of patients treated with belantamab mafodotin in DREAMM-7 and DREAMM-8, respectively, most commonly blurred vision (66% and 79%), dry eye (51% and 61%), foreign-body sensation (44% and 61%), eye irritation (43% and 50%), photophobia (47% and 44%), and eye pain (32% and 33%) [7, 8]. These adverse events are a known class effect of ADCs containing monomethyl auristatin F [9, 10], and are thought to be due to corneal epithelial cell apoptosis following internalization of belantamab mafodotin [11]. In DREAMM-7 and DREAMM-8, OAEs were managed by dose modifications and frequent monitoring, and most events were resolved [7, 8]. Importantly, dose modifications did not appear to affect efficacy outcomes [12, 13].

Based on the results of the DREAMM-7 and DREAMM-8 trials, belantamab mafodotin combinations (with bortezomib and dexamethasone, and with pomalidomide and dexamethasone) have been approved by the Medicines and Healthcare products Regulatory Agency in the UK and by the Pharmaceuticals and Medical Devices Agency in Japan for patients with RRMM [14, 15]. These combinations are also currently under consideration for approvals in several other countries, and there is a need for greater clarity on the management of associated OAEs in clinical practice to facilitate confidence in prescribing. The majority of patients treated with belantamab mafodotin in clinical trials experienced OAEs [7, 8]. Because of this, clear guidance is needed to ensure appropriate care by healthcare teams. This article presents recommendations from a Japanese multidisciplinary expert panel for the management of OAEs in patients with RRMM treated with belantamab mafodotin. These recommendations are intended to support and guide healthcare professionals (HCPs) in the identification and management of OAEs, multidisciplinary collaboration, and a patient-centric approach to treatment.

## Methods

### Expert panel selection

The expert panel consisted of seven haematologists/oncologists and two ophthalmologists from Japan. Haematologists/oncologists were identified based on their expertise in MM and invited if they fulfilled at least two of the following criteria: experience in the DREAMM clinical trials or other relevant trials in MM; author of relevant publications; member of relevant societies/study groups; and contributor to relevant disease management guidelines. Ophthalmologists were identified based on their expertise in the management of ocular conditions and were invited if they had experience in the DREAMM clinical trials or if they fulfilled at least two of the following criteria: experience in other clinical trials; author of relevant publications; and member of relevant societies/study groups.

### Development of recommendations

The expert panel participated in two virtual meetings and was responsible for defining the scope, discussing and agreeing on the recommendations, and reviewing the final text. The first meeting was held on 20 December 2024, where the expert panel identified key themes and clinical questions (CQs) to form a framework for the recommendations. The second meeting was held on 29 January 2025, where the expert panel discussed supporting evidence and their clinical experience, with which they developed a set of recommendations for the management of OAEs in patients treated with belantamab mafodotin in Japan.

## Results

Based on the expert panel’s clinical experience and practical considerations, as well as data from the DREAMM-7 and DREAMM-8 trials, the expert panel identified four key themes in the management of OAEs, under which key CQs were defined to guide the recommendations for managing OAEs in the real-world setting in Japan.

### Theme 1: Identification of OAEs associated with belantamab mafodotin treatment

A thorough baseline ocular assessment should be performed by an ophthalmologist prior to initiation of belantamab mafodotin. Further assessments are recommended during treatment, based on the presence or absence of ocular signs and symptoms.

#### Expert recommendations

##### CQ01: What are the best practices for screening and identifying OAEs in patients treated with belantamab mafodotin?

**Figure 1:**
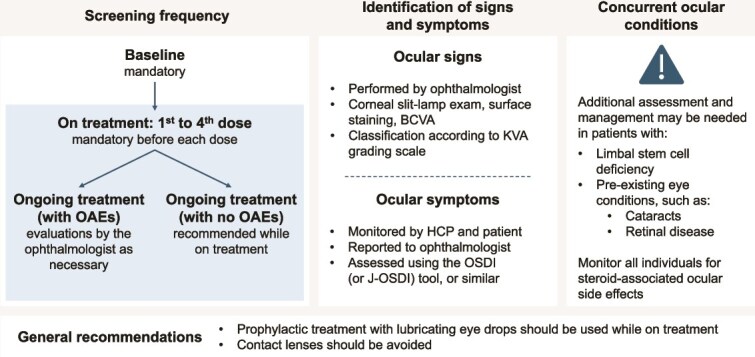
Identifying ocular conditions at baseline and screening for OAEs in response to treatment with belantamab mafodotin. BCVA, best corrected visual acuity; HCP, healthcare professional; J-OSDI, Japanese Ocular Surface Disease Index; KVA, keratopathy and visual acuity; OAE, ocular adverse event; OSDI, Ocular Surface Disease Index.

**Figure 2:**
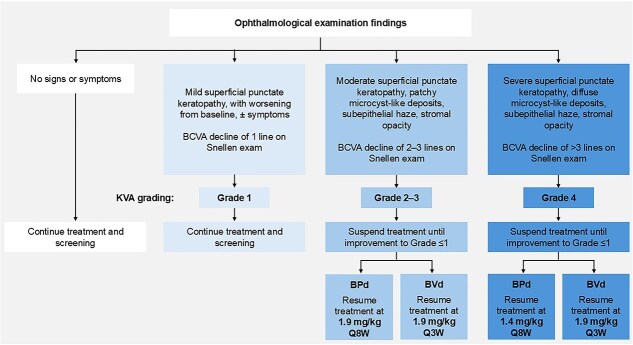
Managing OAEs associated with belantamab mafodotin treatment. BCVA, best corrected visual acuity; BPd, belantamab mafodotin, pomalidomide, dexamethasone; BVd, belantamab mafodotin, bortezomib, dexamethasone; KVA, keratopathy and visual acuity; Q3W, every 3 weeks; Q8W, every 8 weeks.

- Visual acuity testing and corneal examination using a slit lamp to confirm any findings are mandatory before each of the first four doses of belantamab mafodotin, and maintained during treatment (Fig. 1).
  - Intraocular pressure measurements and dilated funduscopic exams should be performed if clinically indicated.
- Appropriate introductory and supplementary materials on potential OAEs with belantamab mafodotin should be provided to the ophthalmologist from the haematologist/oncologist, including clear images and detailed descriptions of corneal adverse events, ideally by grade (refer to CQ03 for details of OAE classification).
- Follow-up from baseline by an ophthalmologist is essential. While the Japan product information for belantamab mafodotin does not provide specific guidance on the frequency of the screening and monitoring of OAEs, the expert panel recommends that patients should be screened for OAEs regularly while on treatment (Fig. 1).
  - Patients with OAEs should be monitored as guided by the ophthalmologist, depending on the patient’s condition, as recommended by the Japanese package insert [16]. Other regional guidelines suggest monitoring every 2 months or more frequently as guided by the ophthalmologist [17].
  - For patients without OAEs, screening is recommended for the duration of treatment with belantamab mafodotin.
- Subsequent treatment with belantamab mafodotin should be determined based on ophthalmological findings (refer to CQ07 and Fig. 2 for specific details on dose modifications for OAEs).
  - Dose modifications depend on the grade of OAE experienced by the patient, as well as the treatment regimen.
- Additionally, it is recommended that patients be interviewed by their haematologist/oncologist regarding any potential adverse events and their interference with activities of daily living, and referred to an ophthalmologist accordingly.
  - Specific and standardized interview questions should be developed to obtain an accurate assessment of subjective symptoms from patients. Existing questionnaires, such as the Japanese Ocular Surface Disease Index (OSDI), should be used to assess the status of dry eye and other subjective symptoms.

##### CQ02: Which key ocular signs and symptoms should be identified and monitored?

- Ocular symptoms frequently reported in clinical trials, such as dry eye, blurred vision, eye pain, reduced vision, and photophobia, should be assessed and monitored by ophthalmologists, as well as managing haematologists/oncologists.
- During treatment, patients should also be monitored for ocular disorders that differ from those caused by belantamab mafodotin, such as infection, cataract, glaucoma, or intraocular haemorrhage.

##### CQ03: How are belantamab mafodotin–associated ocular events classified?

- Belantamab mafodotin–associated ocular events are classified according to the presence or absence of subjective symptoms, which are graded according to the Common Terminology Criteria for Adverse Events (CTCAE) version 5.0 [7, 8, 16].
- Corneal findings are of particular importance, as most subjective symptoms (such as blurred vision and dry eye) are caused by corneal disorders and can be graded according to the Keratopathy and Visual Acuity (KVA) scale [18].
  - Based on the subjective symptom classification table [18], appropriate actions should be considered by comparing baseline assessment (before initiation of belantamab mafodotin) and after Cycle 4 of treatment.

##### CQ04: Are there specific patient subgroups who are more susceptible to ocular events?

- Particular attention should be given to patients with limbal stem cell deficiency, where infiltration of belantamab mafodotin into limbal stem cells may cause further loss of these cells, resulting in invasion of conjunctival epithelial cells through the cornea and coating the cornea with white conjunctiva, leading to vision loss.
  - Patients who may develop limbal stem cell deficiency include patients with congenital disorders (such as aniridia and sclerosing cornea), traumatic disorders (such as thermal or chemical burns of the ocular surface resulting in damage to limbal stem cells), or endogenous disorders (such as Stevens–Johnson syndrome or ocular pemphigoid); limbal stem cell deficiency can also be idiopathic.
- Additional attention should also be given to patients with pre-existing eye conditions, and they should continue their regular visits to their eye clinic independently of their treatment with belantamab mafodotin.
- Monitoring is particularly important in patients who have difficulty perceiving subjective symptoms, such as those who cannot judge subjective symptoms by themselves (for example, elderly patients or patients with cognitive disorders) or those who have cataracts or retinal disease.
- Patients should be advised that wearing contact lenses can also increase the risk of corneal damage and infection.

#### Baseline assessment and monitoring of ocular events

Objective changes to the cornea, with or without subjective symptoms, have been reported in patients treated with belantamab mafodotin, with most events being manageable and resolved [11, 18]. In the DREAMM-7 and DREAMM-8 clinical trials, OAEs of any grade were common in patients treated with belantamab mafodotin (79% and 89%, respectively) [7, 8]. Furthermore, bilateral worsening of visual acuity (20/50 or worse) was seen in 34% of patients with normal baseline assessments in both studies [7, 8]. Median (range) time from the start of treatment to the onset of first worsening of visual acuity (20/50 or worse) was 73.5 (16–753) days and 112 (28–761) days in DREAMM-7 and DREAMM-8, respectively [7, 8].

In clinical practice, because some patients can be diagnosed with an ocular event in the absence of subjective symptoms, while others can present with subjective symptoms before corneal examinations, it is important to consider the commonly reported signs and symptoms and ensure that all HCPs involved in the patient’s care are well educated on identifying these signs and symptoms. Educational materials should be provided to oncologists and ophthalmologists, with advice on techniques for slit lamp examinations, including those required to capture microcysts, along with examples of images of adverse events at different grades.

In the DREAMM-7 and DREAMM-8 clinical trials, subjective symptoms were graded according to the CTCAE [7, 8]. While this was useful in the clinical trial setting, it is often difficult for physicians to interpret and not always used for patient evaluation in real-world clinical practice. Objective findings can be graded using the KVA scale, which was also used in belantamab mafodotin clinical trials [7, 8, 18] and is more practical for clinical practice. However, dry eye and photophobia are commonly occurring subjective symptoms that are not measured with the KVA scale and should be kept in mind when it is used. Symptom-based questionnaires, such as the OSDI [19] or its Japanese version [20], are freely available and reliable, and physicians should consider implementing these questionnaires as part of their practice in patients who are treated with belantamab mafodotin. In addition, the Vision Related Anamnestic tool, a patient-reported questionnaire capturing ocular symptoms and their impact on activities of daily living, has been safe and effective in informing belantamab mafodotin dose modifications in the ongoing BelaRd trial of belantamab mafodotin, lenalidomide, and dexamethasone in patients with NDMM [21].

### Theme 2: Management and dose modification of belantamab mafodotin treatment for OAEs

#### Expert recommendations

Some OAEs may be managed by modifying the belantamab mafodotin dosage or dosing schedule. However, this should be balanced with the need to maintain MM disease control.

##### CQ05: What are the indicators that necessitate belantamab mafodotin dose modification?

- Dose adjustment, interruption, or discontinuation should be decided based on findings from ophthalmologic examination and OAE grading. However, clinical efficacy, as well as subjective symptoms, should also be considered.

##### CQ06: What are the management options for patients who experience OAEs?

- Objective measures, such as 2-degree progression of corneal findings compared with baseline, and patient complaints, should be considered.
- Prophylactic treatment with preservative-free artificial tears should be administered from Cycle 1 Day 1 until the end of treatment.
  - Lubricating eye drops are recommended.
  - Steroid eye drops may cause disturbance to corneal metabolism and have the potential to promote cataract or glaucoma. They should be used with caution in this setting.
- Extended treatment intervals (every 8 weeks or every 12 weeks) have been shown to reduce the incidence of severe eye damage [12, 13].
- Close collaboration between the haematology/oncology and ophthalmology teams is essential to balancing treatment efficacy and safety.
- It is important to be able to reassure patients about any treatment-related OAEs they experience and explain the importance of continuing treatment once OAE symptoms have resolved. HCPs should also provide an overview of the expected outlook and the washout period following which treatment can be resumed (based on the clinical trial background of frequent treatment discontinuation or dose reduction).

##### CQ07: In which cases should the belantamab mafodotin dose be reduced, and what is the recommended level of dose reduction?

- Regarding dose reduction or extension of the dose interval, the criteria should follow those recommended in the Japan belantamab mafodotin product information, which depends on the treatment regimen [16]. These criteria are summarized in Fig. 2 and Supplementary Table S1. In brief:
  - For mild OAEs (minimal keratitis; no impact on visual acuity), treatment can be maintained.
  - For moderate OAEs (moderate keratitis; some impact on visual acuity), reduce belantamab mafodotin dose by 1 dose level.
  - For severe OAEs (severe keratitis + pain; significant impact on visual acuity), reduce belantamab mafodotin dose by 2 dose levels (when used in combination with pomalidomide and dexamethasone), and consider treatment interruption.
- Consultation with the patient is necessary because the effects of ophthalmological symptoms vary depending on the symptoms of MM, the patient’s condition, and the impact of those symptoms on activities of daily living (e.g. whether the patient’s ability to drive a car is affected).
- Should the patient experience improvement in signs and symptoms of MM, modification of belantamab mafodotin dose and dosing interval can also be considered.

#### Dose adjustments for ocular events

In the DREAMM-7 and DREAMM-8 clinical trials, OAEs were managed with dose modifications or interruption, guided by the KVA scale. Median (range) time from onset of first event to resolution was 64 (8–908) days and 29 (7–196) days, respectively [7, 8]. However, it is important to tailor the dose and/or treatment schedule to each patient in order to maximize treatment effectiveness while minimizing OAEs.

When considering dose adjustments, it is essential to think about the impact of the OAE on the patient’s activities of daily living as well as the clinical benefits the patient has derived from belantamab mafodotin. Recent analyses of the DREAMM-7 and DREAMM-8 clinical trials have determined that among patients who required dose modifications, responses were maintained or deepened following the delay; median PFS in these patients was not impacted compared to the rest of the study population [12, 13].

Although OAEs in the DREAMM-7 and DREAMM-8 clinical trials did not indicate a need for permanent treatment discontinuation [7, 8], some patients may continue to experience a high degree of subjective symptoms on resuming treatment, even following an appropriate treatment break. While recurrences of corneal examination findings were common in the DREAMM-7 and DREAMM-8 clinical trials, the duration of each occurrence was consistent and predictable (median 12–13 weeks in patients with no bilateral best corrected visual acuity change to ≥20/50; 11–16 weeks in patients with changes from ≥20/50 to <20/200) [22]. Furthermore, most (995/1192; 83%) grade ≥ 2 occurrences had resolved by data cutoff, and the unresolved occurrences were in patients who were still on treatment/in follow-up (12%) or those who had died or withdrew consent before resolution could be documented (5%) [22]. These factors should be considered together with the clinical benefit of belantamab mafodotin for the patient before continuing treatment.

In addition to dose adjustments, prophylactic or reactive treatments may also help prevent or reduce OAEs. In the DREAMM-7 and DREAMM-8 clinical trials, patients were advised to use preservative-free artificial tears during treatment, and cooling eye masks could be applied during the infusion [7, 8].

### Theme 3: Multidisciplinary collaboration for effective management of ocular events

**Figure 3:**
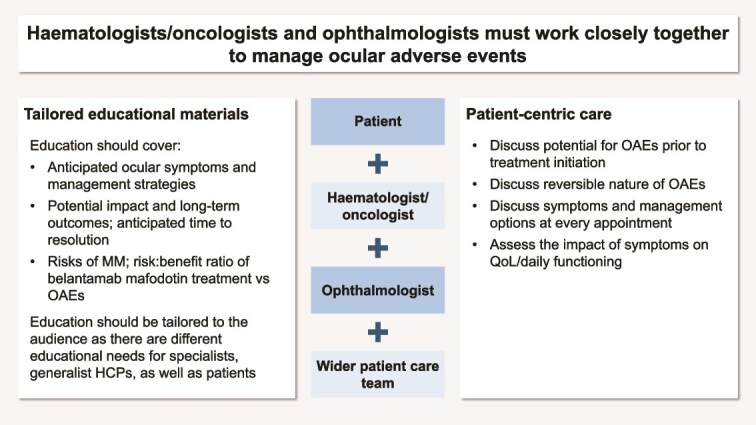
Multidisciplinary collaboration and patient-centred approaches for managing OAEs. MM, multiple myeloma; OAE, ocular adverse event; QoL, quality of life.

Multidisciplinary collaboration is essential to provide optimized care for patients receiving belantamab mafodotin, by facilitating timely exchange of information and expertise (Fig. 3).

#### Expert recommendations

##### CQ08: What are the key clinical indicators of the need for referral to an ophthalmologist?

- Occurrence of OAEs with belantamab mafodotin that affect activities of daily living due to reduced vision indicates a need for referral to an ophthalmologist.
  - In cases of progressive symptoms, close attention should be paid to patients experiencing photophobia, foreign body sensation, and other severe pains.
    ▪ Less commonly, belantamab mafodotin may cause corneal erosion, which may be complicated by bacterial infection, resulting in a bacterial corneal infection constituting an ophthalmological emergency. However, the occurrence of corneal erosion is accompanied by pain in the eye (typically, a persistent foreign body sensation or tingling); therefore, reports of persistent eye pain should prompt referral to an ophthalmologist.
  - It is also necessary to consider the effects of cytomegalovirus retinitis due to immunosuppression, corneal herpes, and steroid-induced cataract and/or glaucoma resulting from steroid use in conjunction with belantamab mafodotin.

##### CQ09: What are the best practices for collaboration between haematologists/oncologists and ophthalmologists?

- An advanced clinical pathway for cooperation with a neighbouring practitioner should be established. If cooperation within the hospital is not possible, it is necessary to establish regional cooperation in advance of using belantamab mafodotin.

##### CQ10: What educational resources and strategies are needed to enhance the understanding of the OAE profile of belantamab mafodotin amongst HCPs?

- Education of HCPs, including nurses and pharmacists, is essential because experience in ophthalmology varies between HCPs with different expertise. Educational materials should be designed for HCPs who may not have much experience in ophthalmology.
- Specifically, ophthalmologists should have sufficient knowledge of:
  - How corneal lesions develop, with an understanding that limbal stem cells in particular are sensitive to belantamab mafodotin and are a major cause of corneal damage.
  - Identification and follow-up of superficial corneal erosions; educational materials should provide a clear view of the entire cornea, with a minimum of five to six serial photographs, to show the process from symptom onset to resolution.
  - Identification of microcysts, using the retroillumination method with a slit-lamp microscope to observe the corneal surface; educational materials should provide examples of a severe microcyst and a schematic of the cornea showing the progression of a microcyst from onset to resolution.
- Education on the objective comparison of slit-lamp examination before and after initiation of belantamab mafodotin, including grading of OAEs, is also recommended to enable standardized examination practices.

#### Multidisciplinary collaboration

Where possible, a multidisciplinary team including haematologists/oncologists, ophthalmologists, and the wider patient care team should be established within the treating institution, for the effective and efficient management of OAEs and belantamab mafodotin–related treatment decisions [18]. Given the minimal overlap in expertise between haematologists/oncologists and ophthalmologists, frequent communication and shared educational resources are essential for the smooth running of the multidisciplinary team. Some patients may also have HCPs or other external support systems who participate in their care, and these people should be included as part of the patient’s multidisciplinary team.

The expert panel noted that in some institutions, it may not be possible to establish a multidisciplinary team internally. In such instances, relationships should be established locally and regionally prior to treating patients with belantamab mafodotin.

### Theme 4: A patient-centred approach to the management of OAEs

Given the subjective nature of some OAEs, patients should be educated on and understand the overall benefit–risk profile of treatment with belantamab mafodotin (Fig. 1).

#### Expert recommendations

##### CQ11: What is the patient's main concern regarding OAEs reported with belantamab mafodotin?

- Patients may experience anxiety caused by the impact of reduced vision on daily life, particularly the increased burden on family members due to driving restrictions and difficulty in independently obtaining information through books, television, and devices. This highlights the importance of adequate patient education prior to treatment initiation to reassure patients that they will be supported with coping strategies.
- Patients may also be concerned with the overall change in quality of vision beyond corneal damage with ophthalmological consultation.
  - Adjustment of reading or driving glasses may reduce the increased stress and vision loss due to corneal damage.

##### CQ12: How can physicians effectively communicate the benefits and risks of belantamab mafodotin to their patients in support of informed decision-making?

- Physicians should ensure that patients are well informed before starting treatment by providing appropriate educational materials, such as those that include images allowing the patient to visualize the effects of reduced vision, and explanation of specific symptoms.
- Physicians should consider that complaints of ocular symptoms can also be associated with disease severity of haematological disorders.
  - Patients with serious primary disease tend to complain less about ocular symptoms, while patients whose primary disease is improving may worry more about their eye health.

##### CQ13: What educational resources and strategies are needed to support patient understanding and compliance?

- It is essential to build relationships of trust and open communication with patients in order to promptly detect and respond to OAEs as they appear during treatment.
- Providing patients with educational materials developed by haematologists/oncologists that help them understand the potential OAEs at a glance, as well as the benefits vs risk of treatment, can help reduce anxiety.
- The use of management notes and check sheets, in the form of a symptom diary to record subjective symptoms on a daily basis, can help patients understand their symptoms with peace of mind, even when they are out of sight of the attending physician.
  - Patients should be encouraged to record their subjective symptoms daily and report them at the time of consultation with their physician.

#### Impact of OAEs on quality of life and patient educational needs

OAEs may be associated with a negative impact on activities of daily living and quality of life, such as by limiting the ability to drive and the resulting stress of perceived burden on patients’ family/caregivers [23, 24]. Importantly, patients who had to stop reading or driving while on treatment in the DREAMM-7 and DREAMM-8 clinical trials experienced this for ~11% of their time on treatment, with most patients returning to baseline reading (56/58; 97%) and driving (46/50; 92%) after a median of 3 weeks, indicating minimal impact on daily activities [22].

The panel agreed that patient education on the potential ocular symptoms before starting belantamab mafodotin will help ease concerns and allow patients to plan ahead for support that they may need. Physicians should also educate patients on the benefits and risks of treatment, and reassure patients that OAEs are manageable and unlikely to have long-term effects on their vision [7, 8, 12, 13, 23].

The ongoing BelaRD trial of belantamab mafodotin, lenalidomide, and dexamethasone in patients with NDMM utilized the OSDI to assess the impact of ocular symptoms on vision-related functioning. While many patients reported OAEs, with most being grade 0–2, few patients reported impacts on activities of daily living 'all of the time’ or ‘most of the time’, suggesting that ocular symptoms do not have major impacts on health-related quality of life [24].

## Conclusions

Belantamab mafodotin–based regimens have demonstrated efficacy benefits over current standards of care in patients with RRMM. However, HCPs may require additional guidance and support to manage OAEs, which, although common in patients treated with belantamab mafodotin, are manageable with dose modification and supportive medications. The recommendations in these practice guidelines build on the published clinical trial evidence and practical experience of the expert panel to help Japanese HCPs make informed treatment decisions in the real-world setting.

## Supplementary Material

Japan_belamaf_consensus_Supplementary_Materials_R1_SUBMITTEDhyaf148

## Data Availability

Data sharing is not applicable to this article as no datasets were generated or analysed during the current study.
