# Supplementary material for: Practical guidance on the clinical management of ocular adverse events associated with belantamab mafodotin in patients with relapsed/refractory multiple myeloma: Recommendations from a Japanese expert panel
Source: Jpn J Clin Oncol. 2025 Oct 6;55(12):1349–56. doi: 10.1093/jjco/hyaf148 (PMC12675259; doi:10.1093/jjco/hyaf148)
Supplement: Japan_belamaf_consensus_Supplementary_Materials_R1_SUBMITTEDhyaf148 [file japan_belamaf_consensus_supplementary_materials_r1_submittedhyaf148.docx]

## **Supplementary Materials**

**Supplementary Table S1.** Modifications to belantamab mafodotin dosing as recommended in the Japan product information [1].

| **Belantamab mafodotin dose** | **In combination with bortezomib and dexamethasone** | **In combination with pomalidomide and dexamethasone** |
| --- | --- | --- |
| Usual dose | 2.5 mg/kg Q3W | 2.5 mg/kg for the first dose; 1.9 mg/kg Q4W for subsequent doses |
| 1-step dose reduction | 1.9 mg/kg Q3W | 1.9 mg/kg Q8W |
| 2-step dose reduction | N/A | 1.4 mg/kg Q8W |

Q3W, once every 3 weeks; Q4W, once every 4 weeks; Q8W, once every 8 weeks.

**Reference**

1. GSK. Blenrep for I.V. infusion. <https://pins.japic.or.jp/pdf/newPINS/00071699.pdf> (1 July 2025, date last accessed).
